# Serum bactericidal assay for the evaluation of typhoid vaccine using a semi-automated colony-counting method

**DOI:** 10.1016/j.micpath.2016.05.013

**Published:** 2016-08

**Authors:** Mi Seon Jang, Sushant Sahastrabuddhe, Cheol-Heui Yun, Seung Hyun Han, Jae Seung Yang

**Affiliations:** a Clinical Immunology, Sciences Unit, International Vaccine Institute, Seoul, Republic of Korea; b Program Management Unit, International Vaccine Institute, Seoul, Republic of Korea; c Department of Agricultural Biotechnology and Research Institute for Agriculture and Life Sciences, Seoul National University, Seoul, Republic of Korea; d Department of Oral Microbiology and Immunology, DRI, and BK21 Plus Program, School of Dentistry, Seoul National University, Seoul, Republic of Korea

**Keywords:** *Salmonella enterica* serovar Typhi, Typhoid vaccine, Serum bactericidal assay, Functional antibody, anti-Vi IgG

## Abstract

Typhoid fever, mainly caused by *Salmonella enterica* serovar Typhi (*S.* Typhi), is a life-threatening disease, mostly in developing countries. Enzyme-linked immunosorbent assay (ELISA) is widely used to quantify antibodies against *S.* Typhi in serum but does not provide information about functional antibody titers. Although the serum bactericidal assay (SBA) using an agar plate is often used to measure functional antibody titers against various bacterial pathogens in clinical specimens, it has rarely been used for typhoid vaccines because it is time-consuming and labor-intensive. In the present study, we established an improved SBA against *S.* Typhi using a semi-automated colony-counting system with a square agar plate harboring 24 samples. The semi-automated SBA efficiently measured bactericidal titers of sera from individuals immunized with *S.* Typhi Vi polysaccharide vaccines. The assay specifically responded to *S.* Typhi Ty2 but not to other irrelevant enteric bacteria including *Vibrio cholerae* and *Shigella flexneri*. Baby rabbit complement was more appropriate source for the SBA against *S.* Typhi than complements from adult rabbit, guinea pig, and human. We also examined the correlation between SBA and ELISA for measuring antibody responses against *S.* Typhi using pre- and post-vaccination sera from 18 human volunteers. The SBA titer showed a good correlation with anti-Vi IgG quantity in the serum as determined by Spearman correlation coefficient of 0.737 (*P* < 0.001). Taken together, the semi-automated SBA might be efficient, accurate, sensitive, and specific enough to measure functional antibody titers against *S.* Typhi in sera from human subjects immunized with typhoid vaccines.

## Highlights

• Improved SBA against *S.* Typhi was developed using a colony counting system.
• The improved SBA was specific to *S.* Typhi but not to other gram-negative bacteria.
• There was a good correlation between SBA and anti-Vi IgG titers in vaccinee’s sera.
• This SBA would be useful for the clinical immuno-monitoring of typhoid vaccines.

## 1 Introduction

Typhoid fever is a gastrointestinal infectious disease caused by *Salmonella enterica* serovar Typhi that is transmitted through the ingestion of contaminated food or water. Risk factors for the disease are high in developing countries due to poor hygiene and sanitation, and the global burden of typhoid fever in 2010 was estimated at 26.9 million cases [1]. Typhoid fever is treated with antibiotics but it has become complicated by the emergence of multi-drug resistant strains of *S.* Typhi [2]. Moreover, the average cost of medical care for typhoid fever is estimated at $4500 per patient in the United States [3]. Thus, reasonably-priced vaccine would be a cost-effective approach to prevent typhoid fever [4].

Currently, two licensed typhoid vaccines, the oral live attenuated *S.* Typhi Ty21a and the parenteral Vi capsular polysaccharide (PS), are known to be safe and efficacious for people aged over 2 years [5]. These two vaccines offer similar levels of protection against typhoid fever and showed similar typhoid-specific humoral responses to Vi and Ty21a in field trials [6]. Although both vaccines confer protective immunity after the vaccination, booster immunization is recommended every 3 years and neither is effective nor licensed for the use in children under 2 years of age [7]. Thus, to overcome these limitations, attempts to develop next-generation typhoid vaccines that confer higher immunogenicity and long-lasting protective immunity in all age groups have been made. Several Vi-conjugated vaccines are under development, including a Vi-diphtheria toxoid [8], Vi-tetanus toxoid [9], Vi-CRM_197_ [10], [11], and live attenuated *S.* Typhi, CVD 909 [12], [13].

For evaluation of typhoid vaccines, a passive hemagglutination assay has been previously used to quantify anti-Vi antibodies in serum after the vaccination [14], [15]. However, it is rarely used due to tedious steps including a requirement for pre-absorption of test sera with sheep erythrocytes [16]. Enzyme-linked immunosorbent assay (ELISA) provides more practical tool to determine serum antibodies against *S.* Typhi in clinical trials [17], [18] but it does not assess functional antibody levels. The serum bactericidal assay (SBA) measures functional *S.* Typhi-specific antibodies capable of complement-mediated bacterial killing. This has been accepted as an *in vitro* surrogate assay for the evaluation of immunogenicity of bacterial vaccines against cholera [19] and meningococcal disease [20] due to its close correlation with protection. Several factors including source and quantity of exogenous complements, bacterial strain, test sera, and antigen expression in target bacteria are important for obtaining reliable SBA results. However, conventional SBA is not appropriate for testing a large number of samples because it is time-consuming and labor-intensive. Here, we describe a simple and convenient SBA against *S.* Typhi using a semi-automated colony-counting system that has been developed for the measurement of cholera [19] and pneumococcal vaccine-induced functional antibody responses [21].

## 2 Materials and methods

### 2.1 Bacteria and reagents

*S.* Typhi Ty2, *S.* Typhi Ty21a, *S.* Paratyphi A, and *S.* Typhimurium were obtained from the American Type Culture Collection (Manassas, VA). *Vibrio cholerae* O1 El Tor Inaba (strain T19479) and *Shigella flexneri* 5a M90T were kindly provided by Prof. Jan Holmgren (University of Gothenburg, Sweden) and Prof. Dong Wook Kim (Hanyang University, Ansan, Korea), respectively. Luria-Bertani (LB) broth and agar were purchased from Conda (Madrid, Spain) and Junsei (Tokyo, Japan), respectively. 2,3,5-triphenyl tetrazolium chloride (TTC) and 4-nitrophenyl phosphate disodium hexahydrate were purchased from Sigma-Aldrich (St. Louis, MO). Baby rabbit (3- to 4-week-old) and adult rabbit (8- to 12-week-old) complements were purchased from Pel-Freez Biologicals (Rogers, AR). Guinea pig and human complements were purchased from Rockland (Gilbertsville, PA) and Valley Biomedical (Winchester, VA), respectively. All complements were stored in aliquots at −80 °C until used. Phosphate-buffered saline (PBS) was purchased from Gibco-BRL (Gaithersburg, MD) and used for serum dilution.

### 2.2 Serum samples

Human sera were randomly chosen from healthy volunteers who had received the Vi PS vaccine during clinical trials and convalescent sera were obtained from cholera and typhoid patients [22], [23]. Use of these samples was approved from the institutional review board of the International Vaccine Institute. The clinical sera, collected prior to and 4 weeks after the vaccination, were examined for SBA and antibody titers. To inactivate complements in the serum, human serum was heated at 56 °C for 30 min before use. Inactivated serum samples were kept at 4 °C until used.

### 2.3 Semi-automated SBA

The original SBA was kindly provided by Dr. Beth Kirkpatrick (University of Vermont, Burlington) and modified by using an automated colony-counting system as previously described [19]. In brief, each serum sample in triplicate was serially-diluted seven times with PBS in two-fold increments. Each well of a 96-well plate (Nunc, Roskilde, Denmark) was filled with 50 μl of serum sample. A single colony of *S.* Typhi Ty2 grown on an LB agar plate was inoculated and cultured in 5 ml of LB broth overnight at 37 °C with shaking. Cultured bacteria were harvested by centrifugation and resuspended in PBS. To adjust the bacteria to 7 × 10^3^ CFU/ml, bacteria were further diluted with PBS, and a 50 μl mixture of bacteria and 10% baby rabbit complement was added to the 96-well microtiter plate containing 50 μl of 2-fold-serially-diluted test serum. After incubation for 1 h at 37 °C, 5 μl of reaction mixture was taken from each well and plated at 3 × 8 (24 samples in total) on a square LB agar plate using an eight-channel micropipette to enumerate colonies in triplicate of 8 samples prepared by 2-fold serial dilution. Once the mixtures were absorbed into the bottom agar, the agar plate was overlaid with a top agar (LB medium containing 2% agar and 100 μg/ml of TTC dye). The plates were then incubated at 37 °C overnight and bacterial colonies on the plates were counted using a colony counting system (FluorChem™ IS-9900; Alpha Innotech, San Leandro, CA). Viability of bacteria was calculated by comparing samples to the number of CFU of complement control in the absence of antibody. SBA titer was determined as the reciprocal highest dilution fold that had ≥50% of bactericidal capacity.

### 2.4 Microtiter plate-based SBA

The initial procedures used in the microtiter plate assay were the same as those in the semi-automated SBA as described above with minor modifications in bacterial numbers (1 × 10^6^ CFU/ml). At the end of incubation, 150 μl of LB media were added to each well and the microplate was incubated for an additional 4, 6, 8, or 16 h and the optical density (OD) of the plate was read at 600 nm with a microplate reader (Molecular Devices, Sunnyvale, CA). The microtiter plate assay against *V. cholerae* was performed as previously described [19].

### 2.5 ELISA

A microwell plate (Nunc) was coated with 2 μg/ml of Vi PS and incubated at 37 °C overnight with gentle shaking. The plate was washed six times with a washing buffer containing 0.85% NaCl and 0.1% Brij 35. To prevent non-specific binding, 200 μl of 1% bovine serum albumin (BSA) in PBS was added and incubated for 1 h at 37 °C with shaking. After washing the plate, 100 μl of pre-diluted sera in 1% BSA/0.1% Brij 35/PBS was added and incubated for 1 h at 37 °C. After washing the plate, 100 μl of alkaline phosphatase (AP)-conjugated anti-human IgG or IgM (1:5000) (Jackson ImmunoResearch Laboratories, West Grove, PA) was added and incubated for 1 h at 37 °C. The plate was washed three times with washing buffer. Then, 100 μl of 4-nitrophenylphosphate substrate (1 mg/ml) in 1 M Tris-HCl supplemented with 3 mM MgCl_2_ at pH 9.8 was added and incubated for 30 min at room temperature. Fifty microliters of 3 M NaOH was added to stop the reaction and OD was measured at 405–490 nm using a microplate reader. Endpoint titers were expressed as the last dilution giving an OD 0.1 higher than the background [24], [25].

### 2.6 Statistical analysis

Mean value ± standard deviation (SD) or standard error (SEM) was determined from at least triplicate in each sample [26]. Statistical significance of serum antibody titers and SBA titers between pre- and post-vaccinated groups was determined by a two-tailed Student’s *t*-test. To examine the relationship between SBA titers and anti-Vi IgG or anti-Vi IgM titers, the results were plotted against each other and Spearman correlation coefficient (*r*) and *P* value were obtained using GraphPad Prism 5 software (GraphPad Software, La Jolla, CA).

## 3 Results

### 3.1 Semi-automated SBA against *S.* Typhi

**Fig. 1:**
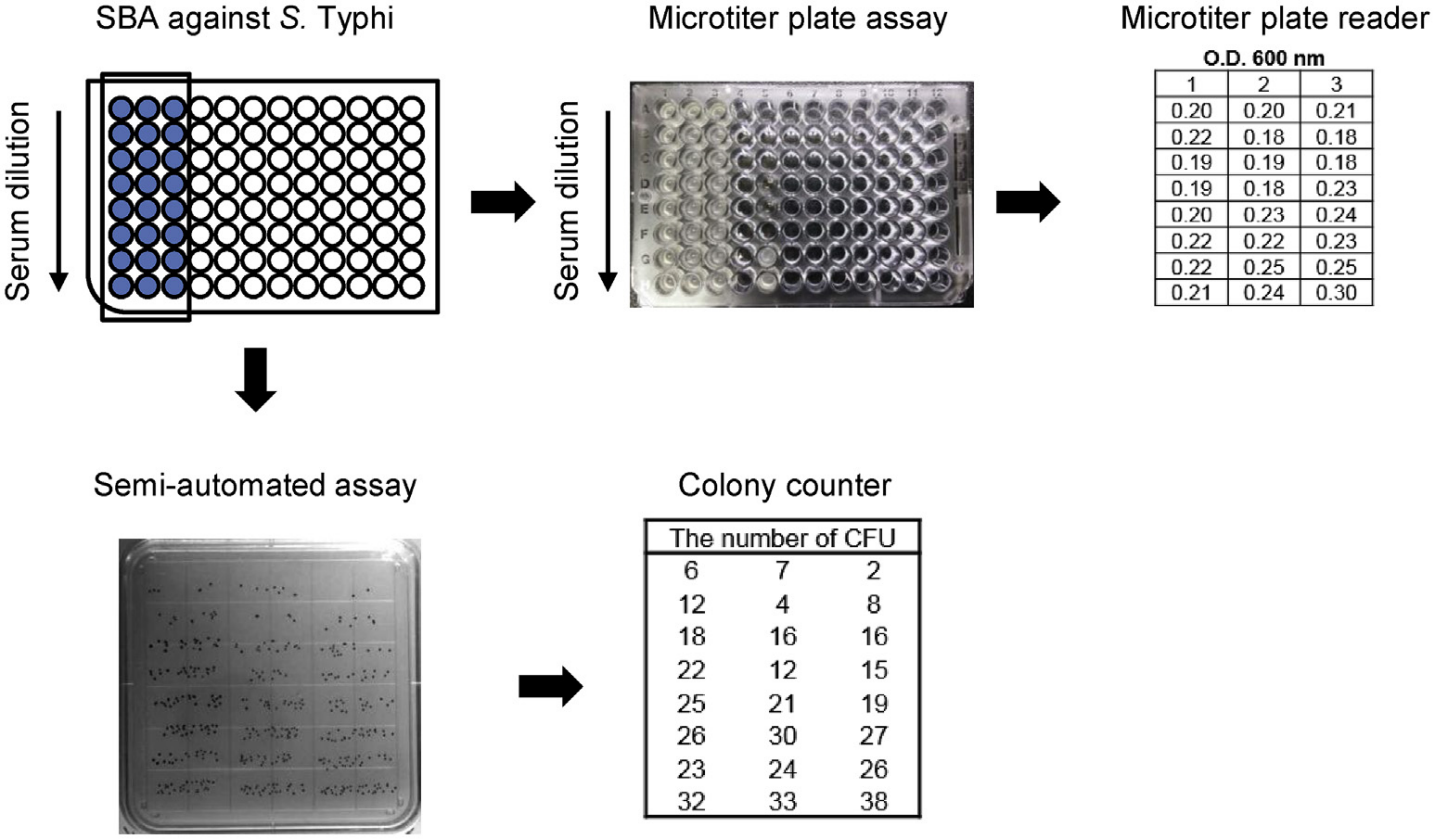
Schematic diagram for the procedure of semi-automated SBA against *S.* Typhi. Human serum from volunteer immunized with Vi PS vaccine was tested for bactericidal activity against *S.* Typhi Ty2 using microtiter plate or semi-automated bactericidal assay. Optical density (O.D.) at 600 nm of a microtiter plate and the number of colony forming unit (CFU) of a square agar plate were measured in triplicate.

Since the conventional SBA against *S.* Typhi based on agar plating method is labor-intensive, we designed a simple microtiter plate assay analogous to one developed for *V. cholerae* [27]. Human convalescent sera from cholera patients showed that bactericidal activity against *V. cholerae* was inversely proportional to the dilution fold of serum, suggesting that SBA titer could be determined by a microtiter plate reader (*data not shown*). However, human anti-Vi serum failed to show SBA titer against *S.* Typhi. Therefore, we used a square agar plate with semi-automated colony-counting system to improve the SBA against *S.* Typhi as previously described [19]. After *S.* Typhi Ty2 was incubated together with serially-diluted sera and 10% baby rabbit complement, 5 μl of reaction mixture from each well was plated in a 3 × 8 pattern (24 samples total) on a square plate. The results showed that the number of colonies was increased proportionally by serum dilution and could be easily counted by using automated colony-counting system (Fig. 1).

### 3.2 Serum from healthy human volunteer immunized with Vi PS vaccine showed specific bactericidal activity against *S.* Typhi Ty2

**Fig. 2:**
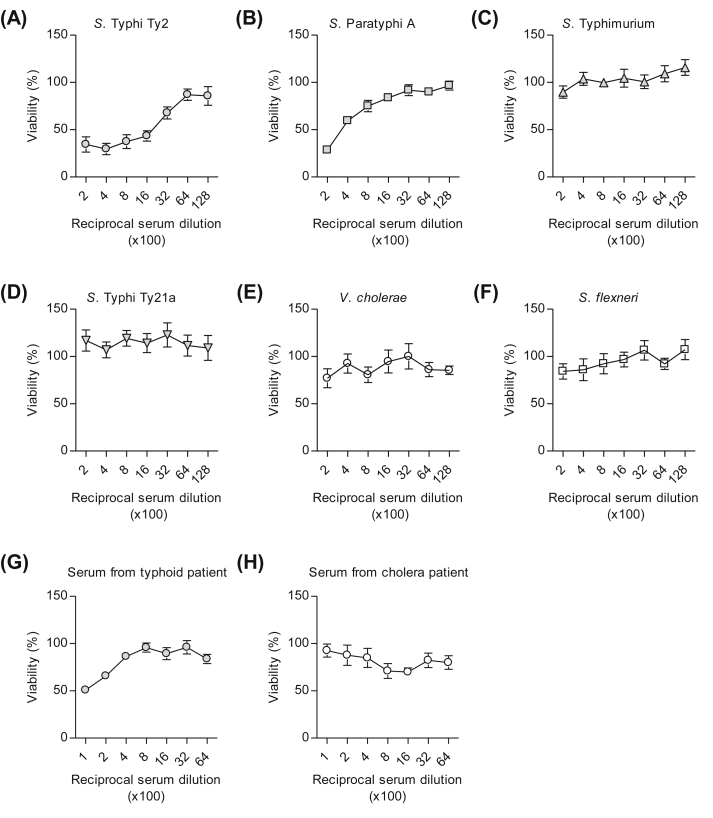
Sera from volunteers immunized with typhoid vaccine specifically inhibit the growth of *S.* Typhi. Human sera from volunteers administered with Vi PS vaccine were tested against various enteric bacteria including (A) *S.* Typhi Ty2, (B) *S.* Paratyphi A, (C) *S.* Typhimurium, (D) *S.* Typhi Ty21a, (E) *V. cholerae,* and (F) *S. flexneri* by the semi-automated bactericidal assay. Human convalescent sera obtained from typhoid (G) and cholera (H) patients were tested against *S.* Typhi Ty2 as the positive and negative control, respectively. Baby rabbit complement (10%) was used in the assay against all bacteria except *S.* Typhi Ty21a (2.5%). Each value represents mean ± SEM of three independent experiments.

To examine specificity of the assay, various enteric gram-negative bacteria such as *V. cholerae*, *S. flexneri*, and *Salmonella* spp. including *S.* Typhi Ty2, *S.* Paratyphi A, and *S.* Typhimurium were tested in the presence of 10%, or 2.5% for *S.* Typhi Ty21a, baby rabbit complement at which bacterial growth for all strains had not been affected by complement’s own activity. The serum from individual immunized with Vi PS showed a marked ability to inhibit the growth of Ty2 more than 50% until reaching 1:1600 dilution while growth of *V. cholerae* and *S. flexneri* was not affected (Fig. 2A, E, and F). Bactericidal activity against *S.* Typhi Ty2 was also observed in the human convalescent sera from typhoid patients but not from cholera patients (Fig. 2G and H). In addition, mild bactericidal activity against *S.* Paratyphi A was observed in serum but not against other *Salmonella* serovars such as *S.* Typhimurium and *S.* Typhi Ty21a (Fig. 2B–D). These data suggest that human sera from those vaccinated with Vi PS specifically inhibit the growth of *S.* Typhi Ty2 in the semi-automated SBA and therefore, this assay can be used for evaluation of immunogenicity of typhoid vaccines.

### 3.3 Baby rabbit complement is appropriate for SBA against *S.* Typhi Ty2

**Fig. 3:**
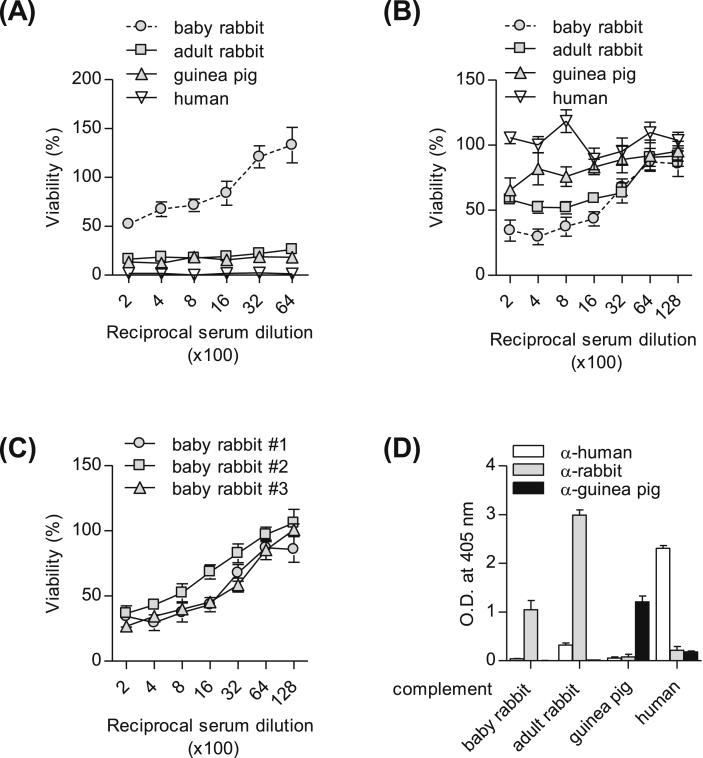
Baby rabbit complement is appropriate for SBA against *S.* Typhi Ty2. (A–C) Sera from volunteers administered with Vi PS vaccine were tested with various mammalian complements. SBA was performed using (A) 10% of each complement, (B) 5% of each complement except baby rabbit (10%), or (C) 10% of baby rabbit complement from three different batches. Each value represents mean ± SEM of three independent experiments. (D) Anti-Vi IgG antibodies in each complement were measured using AP-conjugated anti-human, -rabbit or -guinea pig IgG. Values are mean ± SD of triplicates from each group.

Since complement is a key factor in the SBA and often affects stability of the assay [28], [29], appropriate complement should be selected for the evaluation of bactericidal activity in serum. To examine optimal complement for SBA against *S.* Typhi Ty2, we tested the activities of complements derived from baby rabbit, adult rabbit, guinea pig, and human in the SBA. Growth of Ty2 was proportionally increased by diluting serum in the presence of 10% baby rabbit complement while complements from adult rabbit, guinea pig, and human completely inhibited bacterial growth in a serum-independent manner (Fig. 3A). Therefore, we examined the minimal inhibitory concentration of complements for determining the susceptibility of bacteria to each complement. Since 10% baby rabbit complement and 5% of other types of complement did not affect the growth of Ty2 (*data not shown*), those were tested as complement source. As shown in Fig. 3B, post-immune serum showed typical bactericidal kinetics when baby rabbit complement was used, followed in order by adult rabbit and guinea pig complements; however, post-immune serum did not exhibit bactericidal activity in the presence of human complement. Next, we tested baby rabbit complement from three different batches to assess lot variation on bactericidal activity in serum. Three batches of baby rabbit complement exhibited similar patterns of bacteria-killing capacity in human serum (Fig. 3C). To understand why SBA was more specific to post-immune serum in the presence of baby rabbit complement, we measured Vi-specific IgG levels in various complements. As shown in Fig. 3D, baby rabbit complement had lower anti-Vi IgG antibody levels than other complements derived from human and adult rabbit. These results indicate that baby rabbit complement is appropriate for the SBA against *S.* Typhi Ty2 regardless of batch.

### 3.4 SBA titers highly correlate with the levels of serum IgG against *S.* Typhi Vi

**Fig. 4:**
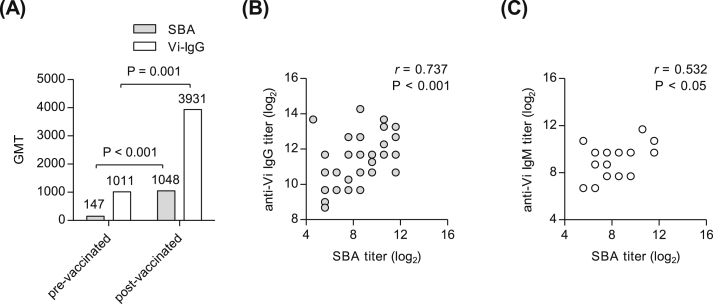
SBA titers are highly correlated with serum anti-Vi IgG titers. (A) Eighteen pairs of serum samples from individuals (pre- and post-vaccination) were analyzed for SBA titer and serum anti-Vi IgG. Geometric mean titer (GMT) of SBA and anti-Vi IgG were analyzed in pre- and post-immunized sera. *P* values were determined using two-tailed Student’s *t*-test for comparison between pre- and post-vaccinated groups. (B, C) Individual SBA titers were plotted against (B) anti-Vi IgG titers against Vi PS in 18 paired serum samples and (C) anti-Vi IgM titers in 10 paired samples. Spearman correlation coefficient (*r*) and *P* value were obtained to examine the relationship between two assays using GraphPad Prism 5 software.

Serum IgG against Ty2 Vi PS using ELISA has been widely used to measure immunogenicity of typhoid vaccine since it is known to be highly correlated with protection from the disease [30], [31]. To compare the differences in anti-Vi IgG and SBA titers after vaccination in human, we measured both titers of pre- and post-vaccinated sera from 18 volunteers administered with Vi PS vaccine (Table 1; Fig. 4). The geometric mean titers (GMTs) for SBA and anti-Vi IgG antibody titers were significantly increased (7-fold, *P* < 0.001, and 4-fold, *P* = 0.001, respectively) after typhoid vaccination (Fig. 4A). As a 4-fold or higher increase in antibody titer after typhoid vaccination is considered as a positive response [17], [32], we analyzed the fold-rise of SBA and anti-Vi IgG titers in post-vaccinated sera from the 18 volunteers. The results revealed that 17 (94%) showed ≥ 4-fold rise of SBA titer as did 11 (61%) of anti-Vi IgG while the fold increase of the two assays was not significantly correlated (Table 1). However, there was a good correlation when SBA titer of each serum sample was compared with anti-Vi IgG of matched sera as assessed by Spearman correlation coefficient, *r* = 0.737 and *P* < 0.001 (Fig. 4B). Next, we measured anti-Vi IgM in 10 paired sera and found *r* = 0.532 and *P* < 0.05 between SBA titer and anti-Vi IgM in sera (Fig. 4C). These results demonstrate that the SBA titer would be another biomarker as well as serum anti-Vi IgG to evaluate typhoid vaccine-induced immunity in clinical study.

## 4 Discussion

SBA has been used to estimate immunogenicity induced by vaccines for protection against cholera, typhoid, and meningococcal disease [19], [33], [34]. However, conventional SBAs for typhoid vaccines are time-consuming and labor-intensive. In the present study, we improved the SBA against *S.* Typhi Ty2 by using a semi-automated colony-counting system for immunological evaluation of typhoid vaccine. The SBA showed reproducible and specific bactericidal activity against *S.* Typhi Ty2 but not against other irrelevant enteric bacteria including *V. cholerae*, *S. flexneri*, and *S.* Typhimurium. In addition, our results demonstrated that baby rabbit complement was appropriate for the assay and superior to complements from other sources including human, adult rabbit, and guinea pig. Moreover, SBA titers of immune sera were highly correlated with serum anti-Vi IgG, which is known to have a close relationship with protective immunity against *S.* Typhi [30], [31]. Therefore, the SBA titer can be used as a biomarker for clinical evaluation of typhoid vaccines.

Our data showed that complement from baby rabbit is the most appropriate source for SBA against *S.* Typhi and the difference in batches did not affect the results. Species-specific variation in bactericidal activity of complements against gram-negative bacteria has long been reported and considered due to diversities in terminal complement components [35], [36], [37]. It is not clear why baby rabbit complement has been used in the bactericidal assay against *Salmonella* spp. [33], [38], [39] and *Neisseria meningitidis* [34], [40]. The growth of Ty2 was significantly inhibited by guinea pig and human complements in a serum antibody-independent manner but not by baby rabbit complement. We speculate that it might be species-specific differences in C3b activation through an alternative pathway. The other possible reason might be attributed to lack of intrinsic bactericidal antibodies, unlike adult vertebrates which have had environmental exposure to ubiquitous bacterial antigens. Indeed, serum IgG and IgM in healthy adults showed bactericidal activity against *S.* Typhimurium but not that in healthy children [41], [42]. This is coincident with our results showing that human and adult rabbit complements had much higher Vi-specific IgG than baby rabbit complement.

Serum from volunteer immunized with Vi PS also showed a mild bactericidal activity against *S.* Paratyphi A but not *S.* Typhi Ty21a or *S.* Typhimurium in the current study. It is probable that *S.* Typhi Ty2 and Ty21a carry O-9 and O-12 while *S.* Paratyphi A has O-1, O-2, and O-12 antigens [43] and *S.* Typhimurium carries O-4, O-5, and O-12 [44]. Thus, *S.* Paratyphi A, Ty21a, and Typhimurium share O-12 antigen of LPS with *S.* Typhi Ty2 but lack Vi PS [6], [45]. Therefore, Vi PS is not expected to generate bactericidal antibody against *S.* Paratyphi A, *S.* Typhi Ty21a, and *S.* Typhimurium. However, our results showed that sera from Vi PS-vaccinated volunteers weakly inhibited the growth of *S.* Paratyphi A. A possible explanation for this may be the contamination with O-12 antigen in the Vi PS during the purification process [6]. Indeed, serum antibodies and specific antibody-secreting cell responses against O-12 antigen were previously detected in a Vi PS vaccination group [6], [46]. This hypothesis was also supported by our results: higher levels of serum IgG against LPS derived from *Salmonella* spp., but not from other enteric bacteria such as *V. cholerae* and *S. flexneri* (*data not shown*). On the other hand, lacking the bactericidal effect of anti-Vi serum on Ty21a might be attributed to the length of the O antigen. The anti-O12 antibody may easily access to LPS of *S.* Paratyphi A because of the short O antigen, while long O antigen on Ty21a and *S.* Typhimurium might interfere with interaction of antibody to bacterial surface [33].

It has been accepted that 3.5 ELISA units of anti-Vi IgG are protective levels in efficacy trials of Vi-rEPA vaccine [30], [31]. SBA titer highly correlating with serum anti-Vi IgG in the present study is suggested to be an alternative surrogate marker for the evaluation of typhoid vaccine efficacy, especially for non-Vi bearing *S.* Typhi such as Ty21a, or LPS- and protein-based typhoid vaccines that is not able to measure anti-Vi IgG level. This finding is also in agreement with an inverse relationship between SBA titers against *S.* Typhi and susceptibility to typhoid [39]; a similar correlation was also observed in cholera [47] and meningococcal diseases [48]. In contrast, a previous study did not show a correlation between SBA titers and anti-Vi PS IgG on the basis of naturally acquired immunity to *S.* Typhi in Nepal, in which *S.* Typhi is endemic [39]. The fact that natural exposure to *S.* Typhi may predominantly induce antibodies against LPS or outer membrane proteins rather than Vi PS may explain the discrepancy. Indeed, anti-Vi antibodies were generated in only 20% of acute typhoid fever cases [49]. Further studies are required to understand the mechanism of protection against *S.* Typhi infection.

Collectively, our results demonstrate that an improved SBA against *S.* Typhi is sensitive, reproducible, cost-effective, and easy to perform with minimal effort using a semi-automated counting system. We anticipate the SBA could be a useful tool to assess typhoid vaccine-induced functional antibody responses.

## Conflict of interest

The authors have no financial conflicts of interest.

## Figures and Tables

**Table 1:** Comparison of SBA titers and anti-Vi IgG and IgM titers in serum from 18 subjects before (Pre-V) and after (Post-V) vaccination with a Vi PS typhoid vaccine.

Serum no.	SBA titers			Anti-Vi IgG titers			Anti-Vi IgM titers		
Pre-V	Post-V	Fold-rise	Pre-V	Post-V	Fold-rise	Pre-V	Post-V	Fold-rise
1	100	400	4	1600	6400	4	800	800	1
2	100	800	8	800	3200	4	100	200	2
3	50	800	16	800	3200	4	100	800	8
4	100	800	8	800	1600	2	400	800	2
5	200	400	2	800	800	1	800	800	1
6	50	1600	32	400	3200	8	1600	3200	2
7	400	3200	8	800	1600	2	800	1600	2
8	200	3200	16	800	3200	4	400	800	2
9	50	200	4	800	3200	4	100	200	2
10	100	400	4	800	1600	2	100	200	2
11	50	400	8	500	19200	38	N.D.	N.D.	
12	200	1600	8	800	3200	4	N.D.	N.D.	
13	200	1600	8	1200	12800	11	N.D.	N.D.	
14	50	400	8	1600	3200	2	N.D.	N.D.	
15	800	3200	4	2400	9600	4	N.D.	N.D.	
16	100	1600	16	1600	4800	3	N.D.	N.D.	
17	800	3200	4	1600	9600	6	N.D.	N.D.	
18	800	3200	4	2400	6400	3	N.D.	N.D.	
GMT	147	1048	7	1011	3931	4	325	650	2

GMT = geometric mean titer, N.D. = not determined.
